# Prevalence of Overweight and Obesity in Children and Adolescents in Eastern Turkey

**DOI:** 10.4274/jcrpe.v2i4.159

**Published:** 2010-11-05

**Authors:** Sevil Arı Yuca, Cahide Yılmaz, Yaşar Cesur, Murat Doğan, Avni Kaya, Murat Başaranoğlu

**Affiliations:** 1 Yüzüncü Yıl University, Faculty of Medicine, Pediatric Endocrinology, Van, Turkey; 2 Yüzüncü Yıl University, Faculty of Medicine, Pediatrics, Van, Turkey; +90 432 216 47 11+90 533 523 90 50sevilyuca@yahoo.comYüzüncü Yıl University, Faculty of Medicine, Pediatric Endocrinology, Van, Turkey

**Keywords:** obesity, child, adolescent

## Abstract

**Objective**: The aim of this study was to estimate the prevalence of overweight and obesity in school children in Eastern Turkey.

**Methods**: This study included 9048 school children aged 6−18 years. The subjects were classified as overweight and obese, according to the International Obesity Task Force.

**Results**: We found prevalence of overweight of 11.1% in the studied population. It was detected that 2.2% of the population in the study was obese; 2.1% of males and 2.3% of females. While the prevalence of obesity was extremely low before 9 ages and after 15, it reached to high values at puberty and just before pubertal period in boys. The prevalence of overweight was higher in girls and reached to peak point at pubertal ages. Generally, the prevalence of obesity and overweight was slightly higher in girls than in boys, although the boys were more obese in prepubertal ages.

**Conclusion**: Overweight and obesity are concerns for children andadolescents in low socio−economic status regions as well.

**Conflict of interest:**None declared.

## INTRODUCTION

Obesity in children and adolescents is a global concern. In developed countries, the prevalence of overweight and obesity in children increased by a magnitude of two to five times in the last quarter of the twentieth century (1, 2, 3, 4, 5). Not only developed countries but also developing countries are adversely affected (6, 7). There are numerous reports in the literature about childhood obesity and its adverse effects on health from different parts of the world (1, 7). Prevalence of overweight and obesity is on the increase also in Turkey, a country, which is undergoing rapid urbanization and changes in nutritional habits.

Reports on prevalence of overweight and obesity from the Eastern parts of Turkey are scarce (8, 9, 10, 11). This study was conducted to obtain data on prevalence of overweight and obesity in school children from the Eastern Anatolian region of Turkey.

## METHODS

This cross−sectional study was carried out between November 2006 and April 2007, and includes representative samples of 6−18 years old children and adolescents attending schools in Van, a city located in the Eastern Anatolian region of Turkey. The entire population of Van city is estimated to be 413 907. The children and adolescents aged 6−18 years constitute 33.3% of this population.

An informed consent form was distributed to the parents one day before the measurements. Participation was voluntary and written informed consent from both parents and children was obtained. The research was approved by the local ethics committee of the Yuzuncu Yil University, Faculty of Medicine as well as from the representative of the Ministry of Education in Van.

A total of 9048 students (4864 males and 4184 females) between the ages of 6 and 18 living in Van city participated in the study.

Measurements of body weight and height were carried out by one trained pediatrician and trained students attending the Medical School of Van Yuzuncu Yil University. Body weight was measured to the nearest 0.1 kg with an electronic scale (Tefal sense, France), with subjects undressed and wearing only underwear. Height was measured to the nearest 1 mm using a portable measuring device (Seca GMTH&CO, 22089 Hamburg, Germany). For height measurements, the subjects had to take off their shoes and they stood erect against a vertical portable scale with their heels, buttocks, scapulae and the back of their heads touching the vertical plane. In the present study, unfortunately pubertal examination was not performed due to socio−cultural reasons.

Body mass index (BMI) was calculated as the ratio of the body weight to the square of body height [BMI=weight (kg)/height(m2)]. Estimation of the prevalence of overweight and obesity was based on the cut−off points of the International Obesity Task Force (IOTF) standards (12).

Information on birth dates was obtained from the parents. If the birth date was not known precisely, these children were excluded. Age groups were defined as year±6 months.

The statistical analyses were performed with SPSS 15.0 software for Windows (SPSS Inc., Chicago, IL, USA). For each survey, means and standard deviations were calculated for each sex. Mean values were compared using the Student’s t test. To define overweight and obesity based on BMI, observed and expected numbers in each category were compared using the x^2^ test. Pearson’s correlation analysis was used to reveal the relationships between obesity and age groups.

## RESULTS

The sample representing the age groups between 6 and 18 years consisted of 9048 subjects (46.2% girls and 53.8% boys). The mean age was 10.3±2.8 years (10.2±2.8 in girls and 10.4±2.9 in boys). Overall prevalence figures for overweight and obesity in this group of Turkish children and adolescents according to gender are shown in Table 1. In the total group, prevalence rates of overweight and obesity were 11.2% (n=1006) and 2.2% (n=198), respectively. These figures were 10.9% and 2.1 % in boys, and 11.4% and 2.3% in girls. The prevalence rates of overweight and obesity in girls were higher than in boys (p=0.001).

Prevalence rates of overweight and obesity according to age and gender are shown in Table 2 and 3 as well as in Figures 1 and 2. In boys, the prevalence of overweight reached to the highest values between ages 10 and 16 years (except for a mild decrease at age 15) with a peak point at age 12 years. In girls, prevalence reached its highest point at age 11 and this high level was maintained until age 16; a decrease occurred at ages 17 and 18, similar to the finding in the boys. Prevalence of overweight was not high in preadolescent ages in either gender, and the lowest value for boys and girls was at ages 9 and 7, respectively.

A comparative evaluation of boys and girls for prevalence of overweight showed that prevalence was higher in boys than in girls at age groups 6,7 and 14 years. In the 12−year age group, prevalence of overweight was similar in girls and boys. Prevalence of overweight was higher in the girls in all other age groups (p=0.001) (Table 2, Figure 2).

The prevalence of obesity was extremely low in the 6−year age group and showed a peak in the age groups 9 and 11 years. Thereafter, prevalence gradually decreased in boys, except for an increase at age 15 years. In girls, prevalence increased with age (except for ages 10 and 11); finally, it reached its highest rate in the 12−13−year age groups, similar to the pattern of prevalence of overweight (Table 3). In boys, prevalence rate was low at ages 14−16, showed an increase at ages 17−18, and decreased after age 15.

In girls, we found a positive and significant correlation between prevalence of overweight and age (OR=0.074/ p=0.001).

**Figures 1:**
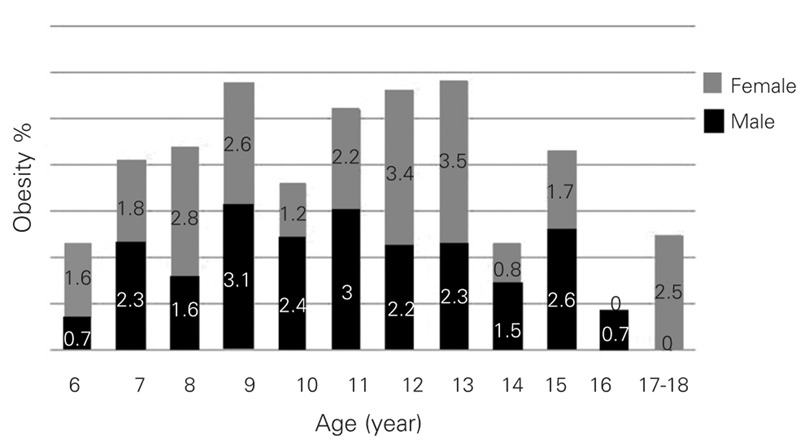
Percentage(%) of obesity in 6−18 years−old children for age and gender

**2:**
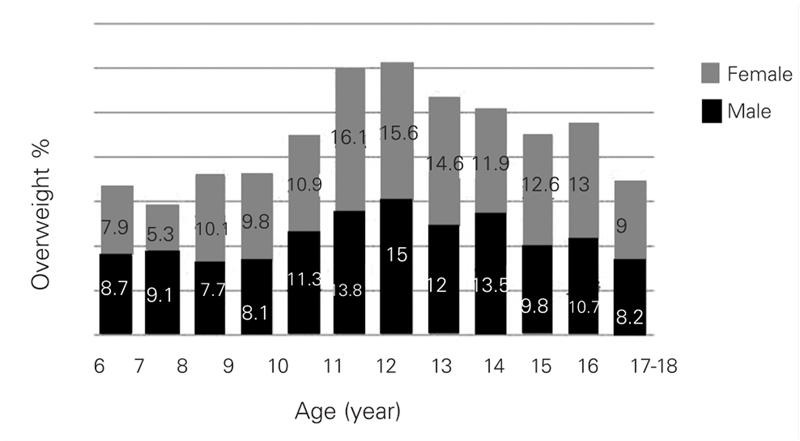
Percentage(%) of overweight in 6−18 years−old children for age and gender

**Table 1:**
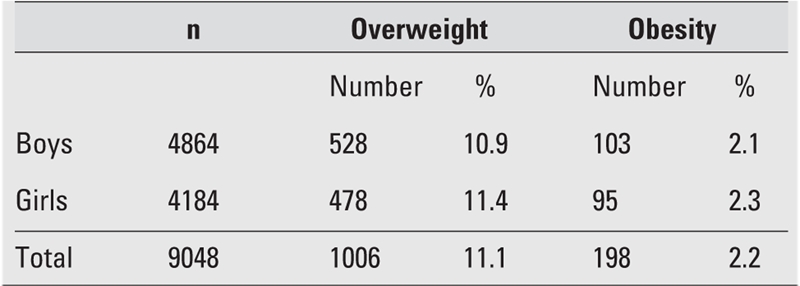
Prevalence of overweight and obesity in boys and girls in the total sample

**Table 2:**
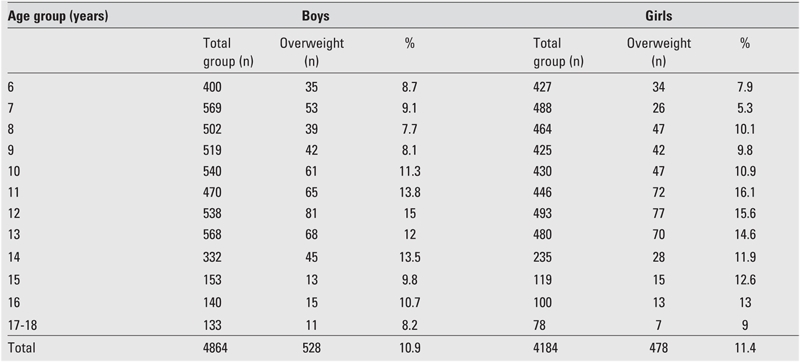
Prevalence of overweight by gender and age

**3:**
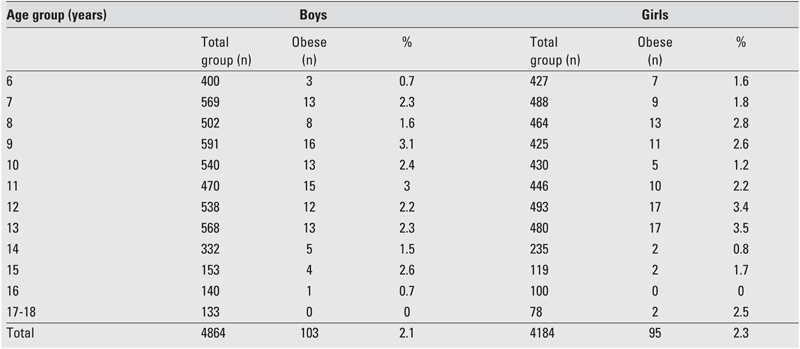
Prevalence of obesity by gender and age

## DISCUSSION

The prevalence of overweight and obesity among children and adolescents worldwide is gradually increasing (13, 14, 15, 16, 17, 18). Although the highest prevalence rates of childhood obesity are observed in developed countries, obesity is also increasing in developing countries (19, 20).

Obesity in adolescence is associated with increased morbidity in adulthood (21, 22, 23, 24). BMI is a sensitive and specific indicator of excess adiposity among children. Overweight is defined as a BMI between the 85th and 95th percentile for age and sex, and obesity−as a BMI greater than the 95th percentile (25, 26, 27). There may be differences in the BMI reference normal standards among different countries, but the IOTF standards are recommended for general use (12).

In our study, the prevalence of overweight was highest at ages 9 and 11 in boys and 12−13 in girls. The prevalence of overweight was low at ages 14−16, but increased again in the older age groups (17−18 years) in girls, whilst prevalence showed a decline after ages 16 in the boys. However, the numbers of subjects may have been too low in the older age groups. In girls, there was a positive and significant correlation between prevalence of overweight and age, but such correlation was not observed in boys.

The prevalence rates of overweight and obesity in our region (Eastern Anatolian) were lower than those previously reported from other areas in Turkey (8, 9). Our data show lower prevalence rates in both genders compared to the rates from regions in the Northern, Southern and Middle parts of Anatolia (in these regions, the overall prevalence of obesity was 6.1%, 3.6%, 4.1%, respectively). Our results are higher than those in boys and almost equal with those in girls from Western Turkey (1.6% in boys, 2.1% in girls) (8, 10).

Comparison of our data with reports from other countries has shown that prevalence of overweight and obesity in our region was lower than those reported from European countries, from US and some Asian countries (2, 5, 7, 13, 28).

In conclusion, we found that the prevalence of obesity was similar in both genders, but the rates showed peaks at different ages (at 11 years in boys and 12−13 years in girls). The prevalence of overweight was higher in girls and showed three peaks (at ages 8−9 years, 11−13 years and 17−18 years). The prevalence of obesity was higher in girls, although the boys were more obese than the girls in some age groups (Figure 1). This is the first report of prevalence of obesity in the Eastern Turkey and our results indicate that overweight and obesity are concerns for children and adolescents in the Eastern Anatolia as well, particularly in adolescent girls.

**Figure 1:**
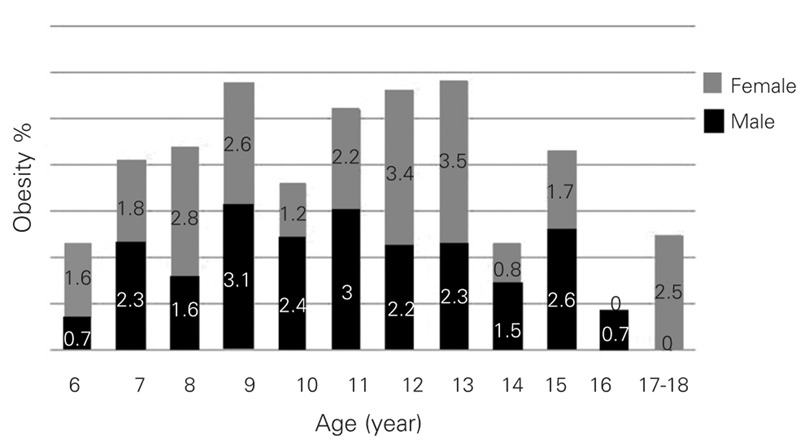
Percentage(%) of obesity in 6−18 years−old children for age and gender
